# Fabrication of Robust Hydrogen Evolution Reaction Electrocatalyst Using Ag_2_Se by Vacuum Evaporation

**DOI:** 10.3390/nano9101460

**Published:** 2019-10-15

**Authors:** Sajjad Hussain, Jinwoong Chae, Kamran Akbar, Dhanasekaran Vikraman, Linh Truong, Bilal Abbas Naqvi, Yawar Abbas, Hyun-Seok Kim, Seung-Hyun Chun, Gunn Kim, Jongwan Jung

**Affiliations:** 1 Graphene Research Institute, Sejong University, Seoul 05006, Korea; shussainawan@gmail.com (S.H.); sbilalnaqvi@gmail.com (B.A.N.); schun@sejong.ac.kr (S.-H.C.); 2 Department of Nano and Advanced Materials Engineering, Sejong University, Seoul 05006, Korea; 3 Department of Physics and Astronomy, Sejong University, Seoul 05006, Korea; sweetdream9234@gmail.com; 4 Department of Energy Science, Sungkyunkwan University, Suwon 16419, Korea; mohkamranakbar@gmail.com; 5 Division of Electronics and Electrical Engineering, Dongguk University-Seoul, Seoul 04620, Korea; v.j.dhanasekaran@gmail.com (D.V.); hyunseokk@dongguk.edu (H.-S.K.); 6 Department of Physics, Khalifa University of Science and Technology, PO Box 127788, Abu Dhabi 147999, UAE

**Keywords:** Ag_2_Se, thermal evaporation, HER, first-principle

## Abstract

Much research has been done on reliable and low-cost electrocatalysts for hydrogen generation by water splitting. In this study, we synthesized thin films of silver selenide (Ag_2_Se) using a simple thermal evaporation route and demonstrated their electrocatalytic hydrogen evolution reaction (HER) activity. The Ag_2_Se catalysts show improved electrochemical surface area and good HER electrocatalytic behavior (367 mV overpotential @ 10 mA·cm^−2^, exchange current density: ~1.02 × 10^−3^ mA·cm^−2^, and Tafel slope: 53 mV·dec^−1^) in an acidic medium). The reliability was checked in 0.5 M sulfuric acid over 20 h. Our first-principles calculations show the optimal energy of hydrogen adsorption, which is consistent with experimental results. The works could be further extended for finding a new catalyst by associating the selenide, sulfide or telluride-based materials without complex catalyst synthesis procedures.

## 1. Introduction

Due to the environmental problems caused by the huge amount of fossil fuels, it is imperative to advance renewable and environmentally-friendly energy sources. Hydrogen generation from water splitting has been considered as a replacement for traditional fossil fuels. Electrochemical water splitting from various catalysts is an efficient way to generate hydrogen [1]. For decades, Pt and noble metals have been used as catalyst materials for hydrogen generation [2,3,4,5,6,7]. From an economic point of view, high prices of those noble metals are a fundamental issue. Therefore, exploring robust, cost-effective, earth-rich, and non-noble metal catalysts to replace Pt is very crucial for hydrogen generation from electrochemical water splitting. Until now, many researchers have made great efforts to search for efficient electrocatalysts, using earth-abundant materials such as metals (Mo, Co, W, Ni, Fe, etc.) along with selenides, sulfides, carbides, phosphides and hybrids [6,7,8]. However, most of the catalysts presented much poor catalytic performance compared to Pt. Besides, many catalysts are synthesized using a complicated preparation procedure to enhance the catalytic properties which, in turn, increases costs.

Since silver (Ag) has the highest electrical conductivity and is rather richer than Pt [9,10], Ag could be considered as a potential candidate. However, the biggest issue of pristine Ag is poor hydrogen evolution reaction (HER) activity, especially in acidic environments. Even though Ag exhibits poor electrocatalytic activity, recently the Ag and S combination (Ag_2_S) exhibited promising, good HER properties [11,12,13,14]. The synergetic chemical coupling behavior between Ag and S elements are assumed to provide the enrichment in the HER activity. Ag_2_S possesses a bandgap of 1.1–2.1 eV with the excellent electrical conductivity, and also theoretically proves its ability for HER reaction [12,13,14]. A few reports for Ag_2_S or Ag_2_S/Ag have been presented [13,15]. Ren et al. [15] observed good HER performance of a porous Ag_2_S/CuS electrocatalyst (200 mV overpotential @ 10 mA·cm^−2^, Tafel slope: 75 mV·dec^−1^). Basu et al. [13] designed Ag_2_S/Ag catalyst (199 mV overpotential, Tafel slope: 102 mV·dec^−1^). Being inspired by excellent electrical properties of Ag_2_S, we combined Ag and Se to implement Ag_2_Se as catalysts. To the best of our knowledge, this is the first report of Ag_2_Se as the electrocatalyst for HER. To overcome previously mentioned obstacles, in this paper, we prepared thin films of Ag_2_Se using a single-step process (vacuum evaporation). Ag_2_Se films with various thicknesses were deposited onto Au/Si in vacuum at room temperature. Ag_2_Se catalysts show good HER properties (367 mV overpotential @ 10 mA·cm^−2^, exchange current density: ~1.02 × 10^−3^ mA·cm^−2^, and Tafel slope: 53 mV·dec^−1^).

## 2. Materials and Methods

### 2.1. Material Synthesis

Highly pure Ag_2_Se powder (99.99%) was procured from Sigma Aldrich (St. Louis, MO, USA). Initially, Au (10–20 nm)/Si substrates were cleaned by deionized (DI) water, methanol, acetone, and isopropyl alcohol. The substrates were loaded on the sample holder and the evaporation chamber was vacuumed by diffusion pump. Ag_2_Se thin films (100–250 nm) were coated at a rate of 1.0 Å·s^−1^. The rate of deposition and film thickness were monitored by a quartz crystal thickness monitor during the film deposition.

### 2.2. Electrochemical Measurements

Electrochemical properties were analyzed using Biologic SP-300 potentiostat. To estimate the HER electrochemical performance for different electro-catalysts, LSV polarization analyses were employed in three-electrode electrochemical cells, in which the silver electrode (Ag/AgCl) used as the reference electrode, the graphite rod as the counterpart electrode, and the Ag_2_Se as the working electrode in 0.5 M H_2_SO_4_ solution. The scan rate was at 10 mV/s. The CV measurements were conducted between +0.1 and −0.4 V vs. Ag/AgCl. LSV curves were recorded using Ag/AgCl, then potentials were changed into the reversible hydrogen electrode (RHE) scale with the support of subsequent calculation: E (sat. Ag/AgCl) + E° (sat. Ag/AgCl) + 0.059 pH. Besides, the working electrode was performed for 20 h to obtain stable polarization curves. EIS studies were performed in the frequencies from 1 Hz to 1 MHz (10 mV). All the polarization measurements were carried out with iR correction using ohmic loss.

### 2.3. Characterizations

Physical morphology and structure of catalysts were studied with scanning electron microscopy (SEM, HITACHI S-4700, Tokyo, Japan). X-ray diffraction (Rigaku, Tokyo, Japan) with Cu-Kα radiation was used to check the film crystallinity. The Raman spectral results were collected using an Ar laser (512 nm) (Renishaw inVia RE04, Gloucestershire, United Kingdom) with a scan speed of 30 s and 1 µm spot size. X-ray photoelectron spectroscopy (XPS, PHI 5000 Versa Probe (25 W Al Kα), Kanagawa, Japan) was used for the chemical compositions.

## 3. Results

**Figure 1:**
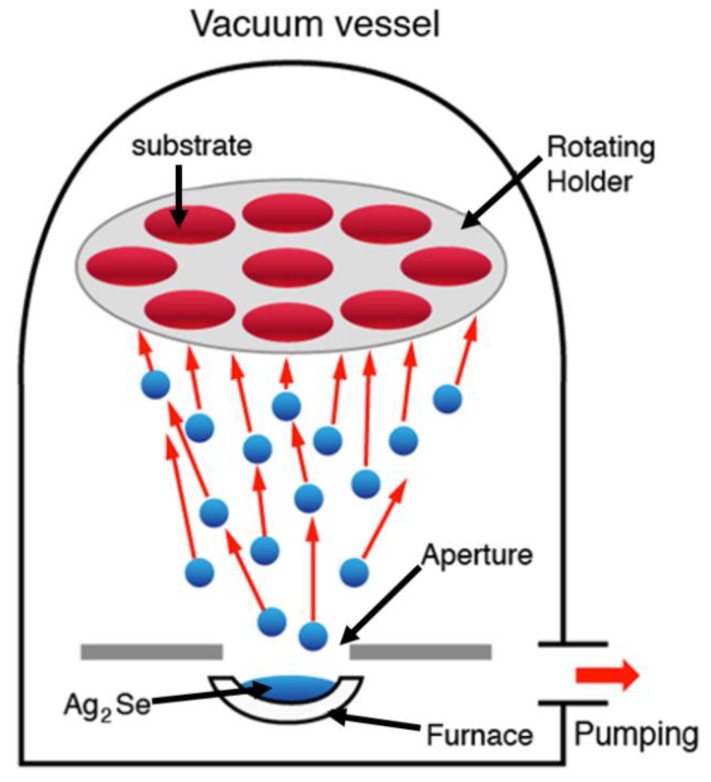
Vacuum thermal evaporator used for the deposition of Ag_2_Se films.

**Figure 2:**
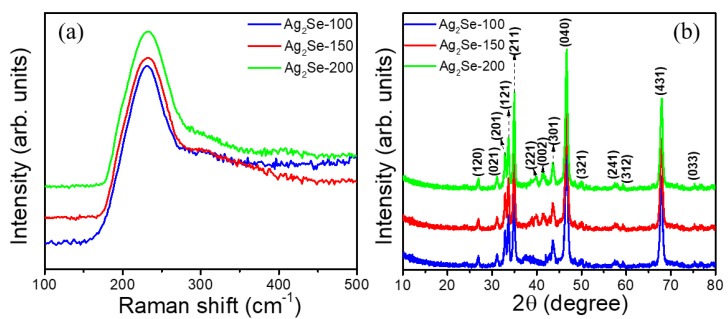
(**a**) Raman and (**b**) XRD profiles of Ag_2_Se films with different thicknesses.

Figure 1 illustrates the schematic diagram of thermally evaporated Ag_2_Se films with various thicknesses (100, 150, and 200 nm), from the bulk polycrystalline Ag_2_Se source. Raman scattering analysis was performed for Ag_2_Se thin films, and their spectra are shown in Figure 2a. The Raman spectra show a rather broad peak @ 232 cm^−1^, which is the characteristics of Ag_2_Se [16,17]. Further, structural characterizations were employed by XRD. From the XRD spectra (Figure 2b), the polycrystalline nature (120), (021), (201), (121), (211), (221), (002), (301), (040), (321), (241), (312), (431), and (033) diffraction lines are observed, which corresponds to the primitive lattice structure of orthorhombic Ag_2_Se. The peak intensities are varied due to different thickness of Ag_2_Se films. The observed peaks clearly follow the standard Ag_2_Se nanocrystal with an orthorhombic system (JCPDS: 89-2591).

**Figure 3:**
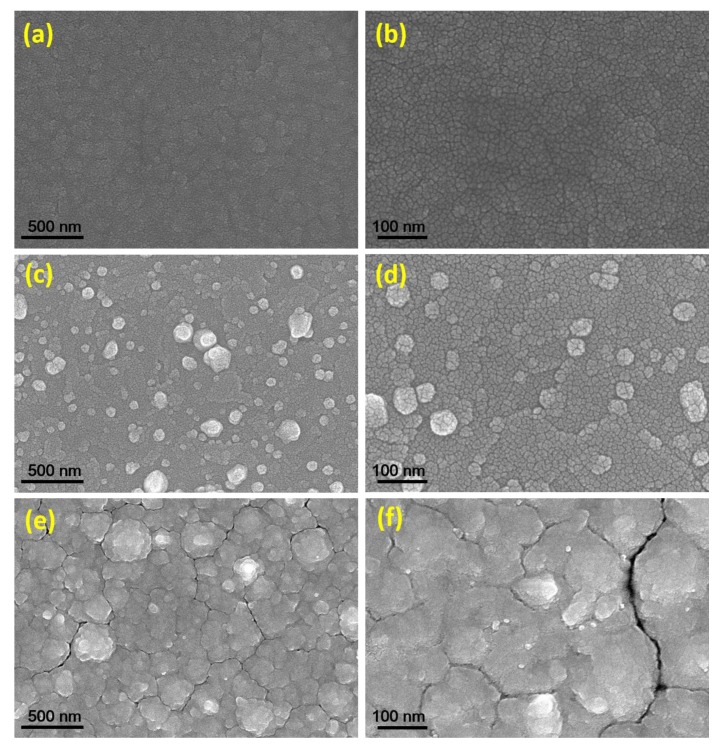
SEM images of the evaporated Ag_2_Se samples. (**a**,**b**) Ag_2_Se-100, (**c**,**d**) Ag_2_Se-150 and (**e**,**f**) Ag_2_Se-200.

SEM examinations were carried out to reveal the surface structure and homogeneity of Ag_2_Se thin films. As shown in Figure 3, the evaporated Ag_2_Se films have uniform morphologies over the surfaces of the film. The smooth surface is found in a 100 nm-thick Ag_2_Se film (labeled as Ag_2_Se-100) due to low film thickness, as shown in Figure 3a,b. For 150 nm-thick-Ag_2_Se (labeled as Ag_2_Se-150) in Figure 3c,d, the film surface is covered with an irregular spatial type of grains. The different sizes of grain clusters are exhibited by agglomerations which tend to form larger size grains due to increased film thickness. For the 200 nm-thick Ag_2_Se film (labeled as Ag_2_Se-200) in Figure 3e-f, the surface of the film consists of different sizes of grain domains with different shapes, possessing pinholes, and hillocks which may affect the conductivity of the film. The existence of Ag and Se stoichiometric ratio, and atomic homogeneity (Ag_2_Se-200) were proved by energy-dispersive X-ray spectroscopy (EDS) (Figure S1, and Figure S2 of Supplementary Materials).

**Figure 4:**
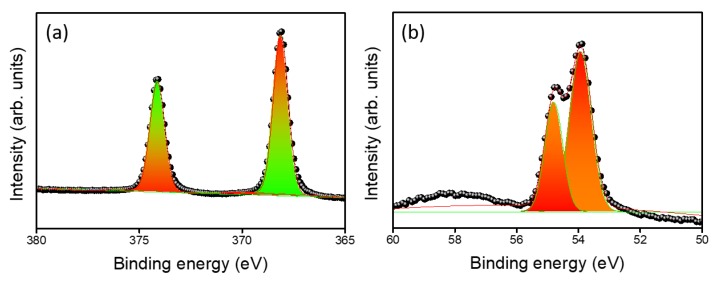
XPS spectra of Ag_2_Se-200 (**a**) Ag 3d and (**b**) Se 2p atom.

The chemical compositions of Ag_2_Se were further investigated by XPS analysis. XPS spectra are shown for Ag_2_Se-200 (Figure 4). For Ag 3d orbitals, as shown in Figure 4a, binding energies at 368 and 374 eV are ascribed to the spin-orbit doublet Ag 3d_5/2_ and Ag 3d_3/2_, respectively [18]. The energy peaks observed at 53.88 and 54.78 eV are assigned to the Se 2p_1/2_ and Se 2p_3/2_ orbital divalent selenide (Se^2−^), respectively, as shown in Figure 4b. The calculated stoichiometric ratio is 2:1 and observed XPS results are coordinated with the literature for Ag_2_Se crystal [18]. The survey spectrum is given to verify all the elements in the Ag_2_Se film (Figure S3).

**Figure 5:**
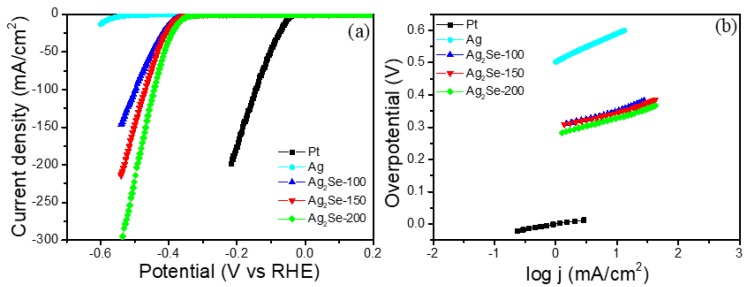
Electrocatalytic hydrogen evolution of different catalysts. (**a**) Polarization curves of commercial Pt, pure Ag (200 nm) and different thickness of Ag_2_Se and (**b**) their corresponding Tafel plots.

The catalytic activity of the Ag_2_Se and Pt electrode for the HER was examined using a three-electrode system in 0.5 M H_2_SO_4_ electrolyte solution (linear sweep voltammetry (LSV), 10 mV/s scan rate). As expected, the commercial Pt wire exhibited very efficient HER activity (54 mV overpotential @ 10 mA·cm^−2^). Ag_2_Se-200 showed 367 mV overpotential @ 10 mA·cm^−2^, which is 15 and 23 mV lower potential than that of Ag_2_Se-100 and Ag_2_Se-150 (Table 1). HER electrocatalytic activities revealed that Ag_2_Se thickness plays a vital role because of the variation of active edges and morphological properties with deposition time. For Ag_2_Se-250 sample, the lower catalytic activity may be due to the surface continuity of the film (Figure S4). Thick films could disturb electrolyte penetration and diminish the role of inherent Ag_2_Se active edges thereby decrease the electrocatalytic activity. A pure 200 nm-thick Ag film (labeled as Ag) exhibited 588 mV at 10 mA·cm^−2^, as shown in Figure 5a. In earlier studies, HER properties were demonstrated for the various layer materials including, bulk and their exfoliated layers of TMD materials such as high-quality monolayer MoS_2(1−x)_Se_2x_ (η_10 mA·cm_^−2^ = 273–300 mV) [19], S-doped MoSe_2−x_ nanotubes and MoSe_2_ nanocaterpillars (η_2 mA·cm_^−2^ = 95–318 mV) [20], tungsten selenide thin films deposited on tungsten foils (η_10 mA·cm_^−2^ = 350 mV) [21], electrodeposition of amorphous cobalt–cobalt oxide/cobalt selenide (CoO_x_–CoSe) composite film on a 3-dimensional porous Ni foam (NF) (η_10 mA·cm_^−2^ = 300–380 mV) [22], and electrodeposited cobalt selenide nanostructures (η_10 mA·cm_^−2^ = 472–481 mV) [23].

The extracted Tafel slope values are 53, 51, 55 and 87 mV·dec^−1^ for Ag_2_Se-200, Ag_2_Se-150, Ag_2_Se-100, and pure Ag, respectively (Figure 5b). We believe that increasing the deposit time would promote the surface-active edge sites and increase the catalytic activity of the electrode. In addition, Ag_2_Se-150 and Ag_2_Se-200 catalysts offer lower Tafel slopes than previous selenide-based HER catalysts, such as CoS_2_-WS_2_ electrocatalysts (66.0–79.6 mV·dec^−1^) [24], cobalt selenide/NiFe layered on exfoliated graphene foil (68–236 mV·dec^−1^) [25], hybrid structure of MoS_2_-WS_2_ (72 mV·dec^−1^) [26], NiSe nanofiber (64 mV·dec^−1^) [27], MoSe_2_/graphene hybrid nanostructures (67 mV·dec^−1^) [28], WSe_2_ and WS_2(1−x)_Se_2x_ (99 and 105 mV·dec^−1^) [29], hybrid structure of MoS_2_/NbSe_2_ nanobelts (79.5 mV·dec^−1^) [30], WS_2(1__−x)_Se_2x_ nanoribbons (68 mV·dec^−1^) [31], WS_2_ nanotube and phosphorous doped WS_2(1−x)_P_2x_ nanoribbon (NR) (83 and 71 mV·dec^−1^) [32]. 

HER kinetics can be processed via a Volmer-reaction (discharge step, Equation (1)) tailed by either an Heyrovsky reaction (ion-atom step, Equation (2)), or combination process (Tafel-reaction, Equation (3)) [33,34].

(1) $$H_{3}O^{+}+e^{-}\to H_{ads}+H_{2}O$$

(2) $$H_{ads}+H_{3}O^{+}+e^{-}\to H_{2}+H_{2}O$$

(3) $$H_{ads}+H_{ads}\to H_{2}$$

The Tafel slope of 51 mV·dec^−1^ for Ag_2_Se-150 signifies that HER occurs through the Volmer–Heyrovsky kinetics, where a rapid protons discharge (Equation (1)) is tailed by a Heyrovsky reaction (Equation (2)) or combination reaction step (Equation (3)) [35,36]. Exchange current density (J_0_), another HER characteristic assessment of electrocatalysts, was obtained by extrapolation of Tafel plot towards cathodic current (Table 1). The reported values of J_0_ for ternary selenide- or sulfide-based catalysts for HER are presented in Supplementary Materials. Cyclic voltammetry (CV) was measured for the double layer capacitances (C_dl_) and the assessment of electrochemically effective surface area (ECSA), assuming that those quantities are directly proportionate [11,37]. The C_dl_ of Ag_2_Se samples are measured by CV at non-faradaic potential regions (Supplementary Figure S5), and is extracted at a given potential (0.24 V vs. RHE) from the differences between cathodic and anodic current density values (∆j = j_a_ − j_c_) against the various scan rates (20–100 mV·s^−1^). Ag_2_Se-150 displays 55.9 μF·cm^−2^ of C_dl_ value and it is greater than that of the Ag_2_Se-100 (41.1 μF·cm^−2^) and Ag_2_Se-200 (50.5 μF·cm^−2^), indicating the propagation of more active edges in the catalyst, resulting in the enhanced catalytic activity. From these capacitance values, the obtained ECSA values are 2.34 cm^2^ (Ag_2_Se-200), 3.19 cm^2^ (Ag_2_Se-150), 2.88 cm^2^ (Ag_2_Se-100) [38]. The results indicate that the Ag_2_Se-150 has the largest ECSA among the three samples.

**Figure 6:**
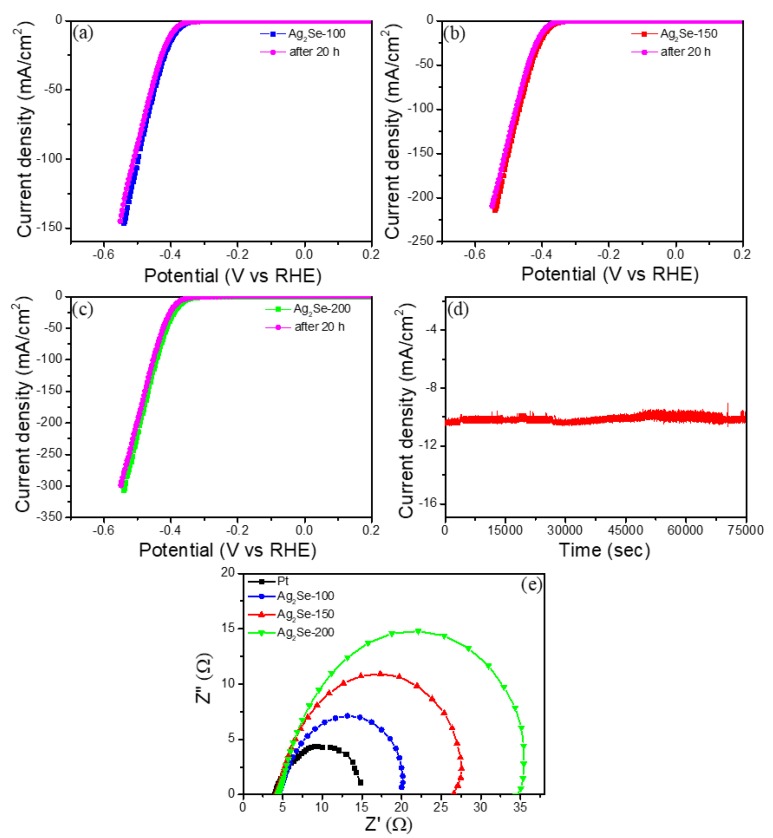
(**a**–**c**) Polarization curves of Ag_2_Se catalysts before and after 20 h HER performance, (**d**) Chronoamperometric responses (*j-t*) recorded for Ag_2_Se-200 at a constant overpotential, (**e**) electrochemical impedance spectroscopy (EIS) spectra for Pt and Ag_2_Se catalysts.

For an efficient HER catalyst, cycling stability is also important. To probe the stability of Ag_2_Se in an acidic medium, an incessant HER cycling test at a static overpotential was conducted. The robustness of Ag_2_Se-200 was further proved by employing continuous hydrogen evolution in 0.5 M H_2_SO_4_ for 20 h, and then, the polarization curve was recorded between −0.6 and 0.2 V vs. RHE. The polarization curves of the three electrodes even after the 20 h successive HER operation, were changed little from their initial curves, as shown in Figure 6a–c. For the chronoamperometric (*j-t*) profile, a static overpotential of 367 mV (Ag_2_Se-200) was used for 20 h, as shown in Figure 6d. The current density exhibits a robust stability performance in a continuous HER operation (over 20 h). Electrochemical impedance spectroscopy (EIS) was carried out to examine the junction reactions and electrocatalyst kinetics of HER process over the frequency range between 1 Hz and 1 MHz. Nyquist plots (Figure 6e) reveal the charge-transfer resistance (R_ct_) values at 16.6 Ω (Ag_2_Se-100), 22.1 Ω (Ag_2_Se-150), and 30.2 Ω (Ag_2_Se-200) for Ag_2_Se catalyst. The small series resistance (4.1–4.5 Ω) for all catalysts confirms the significance of the preparation of high conductive Au substrate, which allows comfort and operative electrical integration that diminishes ohmic losses.

XPS spectra taken after the 20 h continuous HER operation revealed the slight alteration in the Se binding energy spectrum intensity due to the Se active facets contribution to produce and sustain HER properties in Ag_2_Se-200 film (Figure S6), supporting the long-time HER robustness of Ag_2_Se. An SEM image taken after the 20 h stability test is shown in Figure S7.

**Figure 7:**
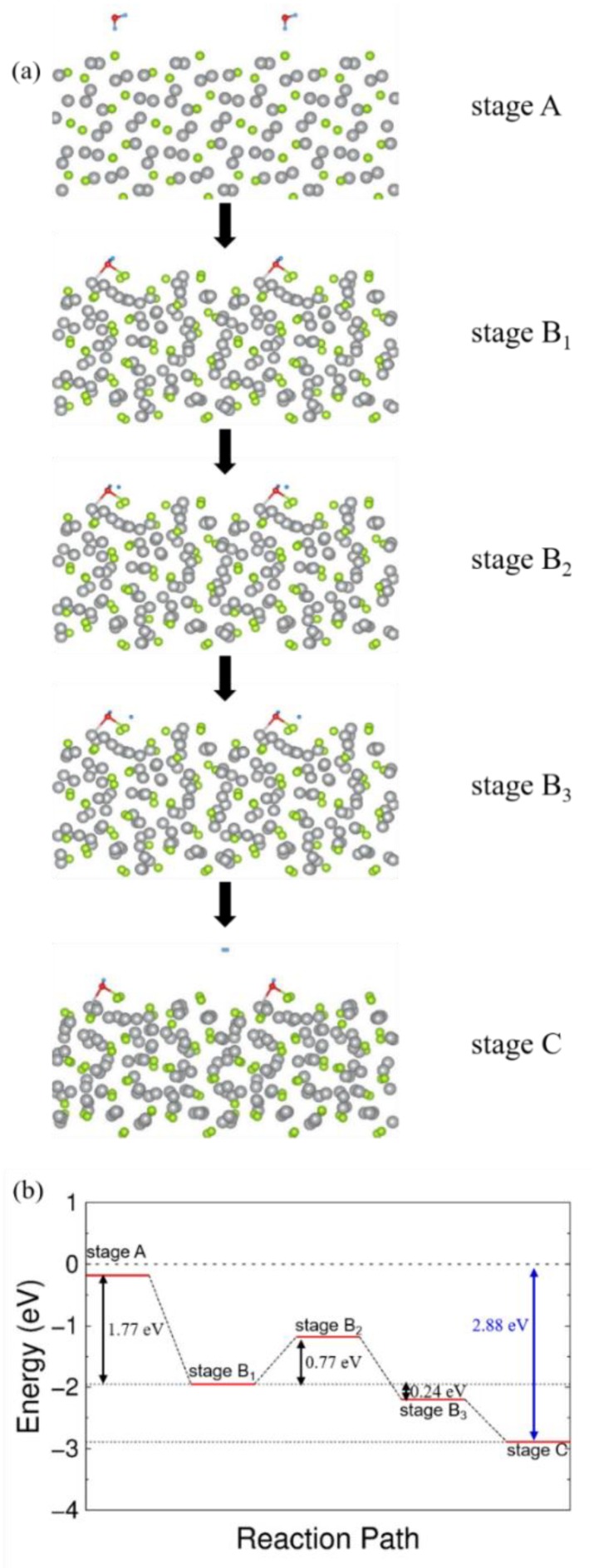
Adsorption process of Ag_2_Se by the DFT calculation. (**a**) At stage A, an H_2_O molecule is adsorbed on the Ag_2_Se surface. At stage B_1_, the water molecule bonds to a selenium atom of the Ag_2_Se surface. At stage B_2_, the water molecule is dissociated into an H atom and a–OH chemical group. At stage B_3_, the separated H atom bonds to an adjacent Se atom. At stage C, H atoms make molecular hydrogen gas. (**b**) The activation and reaction energies in the reaction path.

To validate the capability of Ag_2_Se [39,40] acts as a catalyst for decomposing water molecules, we performed ab initio total-energy calculations based on the density functional theory (DFT) [41] within the generalized gradient approximation [42] for the exchange-correlation functional. The wave functions were extended using a plane-wave basis set [43,44], and the cutoff energy was 600 eV. Various model structures were considered: A water molecule and a hydroxyl (-OH) group were adsorbed on a 2 nm-thick slab of Ag_2_Se. We used the nudged elastic band (NEB) method to calculate the activation energy barrier. Six replicas were chosen, including the initial and final configurations. For a reaction path of decomposition, as shown in Figure 7, we considered several steps (stage A, stage Bi (i = 1, 2, 3), and stage C). At stage A, an H_2_O molecule is located 3.5 Å from the Ag_2_Se surface. Stages B_1_, B_2_, and B_3_ are intermediate configurations, and stage C is the final configuration in which a hydroxyl (-OH) group is adsorbed onto the Ag_2_Se surface. At stage B_1_, the H_2_O molecule is bonded to a Selenium atom in Ag_2_Se. At stage B_2_, the HO-H bond of a water molecule (H_2_O) becomes dissociated. At stage B_3_, the separated H atom hops to an adjacent Se atom and is bonded. We calculated the total energy of each system and found that the reaction energy (E_r_) is −2.88 eV. Here, E_r_ is defined as: Er = E [OH at Ag_2_Se] + 1/2 E [H_2_] − E [H_2_O] − E [Ag_2_Se].

The activation energy barrier is calculated to be 0.77 eV. The activation energy is dependent on the path with initial and final configurations, but we can expect that the actual energy barrier could be less than 1 eV because we did experiments at room temperature. We note that dissociation of HO-H bond of a water molecule (H_2_O) requires 5.03 ± 0.2 eV [45] in the absence of the catalyst. The geometrical information about the absorption process is presented in the Supplementary Figure S8. For the binding of the hydroxyl group, the bond length is slightly longer than the well-known covalent bond lengths [46] of Se and O atoms because the O atom also has weak interaction with a neighbor Ag atom. The covalent radius of Se is 1.2 Å, and that of O is 0.66 Å.

## 4. Conclusions

Ag_2_Se thin films with various thicknesses (100–200 nm) were deposited by a simple thermal evaporation route. The electrochemical surface area values assessment revealed the mechanism of HER electrocatalytic properties in Ag_2_Se thin films. The Ag_2_Se catalysts showed good HER electrocatalytic performances in an acidic medium (367 mV overpotential at 10 mA·cm^−2^, 53 mV·dec^−1^ Tafel slope, and ~1.02 × 10^−3^ mA·cm^−2^ exchange current density). Using first-principles calculations, we investigated the water splitting mechanism for the Ag_2_Se catalysts. The activation energy barrier was estimated to be 0.77 eV. From the observed results, we believe that Ag_2_Se-based catalyst will pave a novel pathway to improve the favorable use as the heterogeneous catalysis for catalytic electrochemical reactions and hydrogen-driven solar energy.

## Figures and Tables

**Table 1:** HER parameters for Ag_2_Se, Ag, and Pt.

Sample	Overpotential (mV vs. RHE) at 10 mA·cm^−2^	Tafel Slope (mV·dec^−1^)	Exchange Current Density (j_0_, mA·cm^−2^)
Pt	54	31	9.86 × 10^−1^
Ag_2_Se-200	367	53	1.02 × 10^−3^
Ag_2_Se-150	382	51	5.12 × 10^−4^
Ag_2_Se-100	390	55	6.45 × 10^−4^
Ag	588	87	1.31 × 10^−5^

## Supplementary Materials

The following are available online at https://www.mdpi.com/2079-4991/9/10/1460/s1, Figure S1. EDS spectrum of elemental composition for a 200 nm-thick Ag2Se film; Figure S2. (**a**) FESEM image of an Ag2Se-200 film and (**b**,**c**) their elemental mapping images of (**b**) Se and (**c**) Ag elements; Figure S3. XPS survey spectrum of an Ag2Se-200 film; Figure S4. (**a**,**b**) FE-SEM image of Ag2Se-250 (**c**) Polarization curves of Ag2Se-200 and Ag2Se-250; Figure S5. Electrochemical cyclic voltammetry curves of as-grown catalysts at different potential scanning rates. (**a**) Ag2Se-100 (**b**) Ag2Se-150 and (**c**) Ag2Se-200; Figure S6. XPS spectra of before and after 20 h HER performance of Ag2Se-200 (**a**) Ag 3d and (**b**) Se 2p orbitals; Figure S7. (**a**,**b**) FE-SEM image of Ag2Se-200 catalyst before and after 20 h HER operation; Figure S8. Geometrical bond distances for the absorption process; Table S1. HER catalytic performances for different electrocatalysts.
