# Supplementary material for: Fabrication of Robust Hydrogen Evolution Reaction Electrocatalyst Using Ag2Se by Vacuum Evaporation
Source: Nanomaterials (Basel). 2019 Oct 15;9(10):1460. doi: 10.3390/nano9101460 (PMC6835317; doi:10.3390/nano9101460)
Supplement: Supplementary file 1 [file nanomaterials-09-01460-s001.pdf]

Electronic Supplementary Information

Fabrication of Robust Hydrogen Evolution Reaction Electrocatalyst using Ag_2Se by Vacuum Evaporation

Sajjad Hussain[a,b], Jinwoong Chae [a,c], Kamran Akbar[d], Dhanasekaran Vikraman[e], Linh Truong[c], Bilal Abbas Naqvi[a,b], Yawar Abbas[f], Hyun-Seok Kim[e], Seung-Hyun Chun[a,c], Gunn Kim[*a,c], and Jongwan Jung[*a,b]

[a]Graphene Research Institute, Sejong University, Seoul 05006, Republic of Korea
[b]Department of Nano and Advanced Materials Engineering, Sejong University, Seoul 05006, Republic of Korea
[c]Department of Physics and Astronomy, Sejong University, Seoul 05006, Republic of Korea.
[d]Department of Energy Science, Sungkyunkwan University, Suwon 16419, Republic of Korea
[e]Division of Electronics and Electrical Engineering, Dongguk University-Seoul, Seoul 04620, Republic of Korea
[f]Khalifa University of Science and Technology, PO Box 127788, Abu Dhabi, UAE
*Correspondence: jwjung@sejong.ac.kr (J.J.); gunnkim@sejong.ac.kr (G.K.)

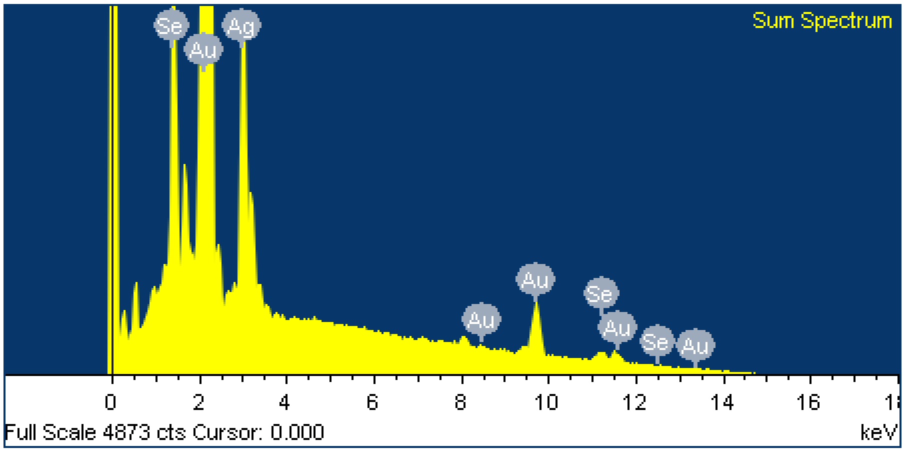

Figure S1. EDS spectrum of elemental composition for a 200-nm-thick Ag_2Se film.

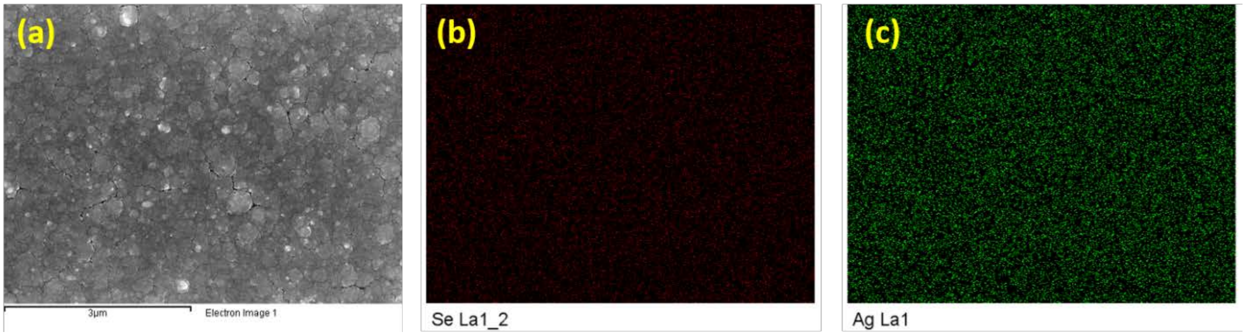

Figure S2. (a) FESEM image of an Ag_2Se-200 film and **(b-c**) their elemental mapping images of (b) Se and (c) Ag elements.

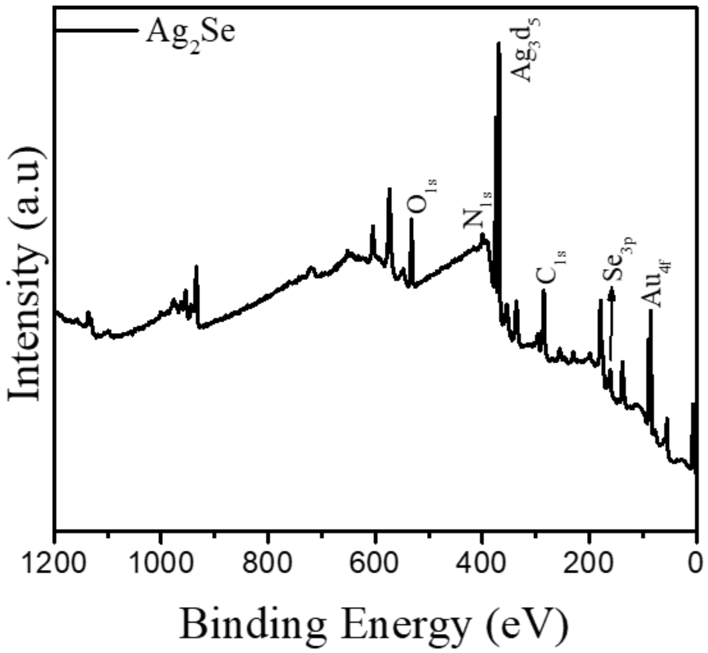

Figure S3. XPS survey spectrum of an Ag_2Se-200 film.

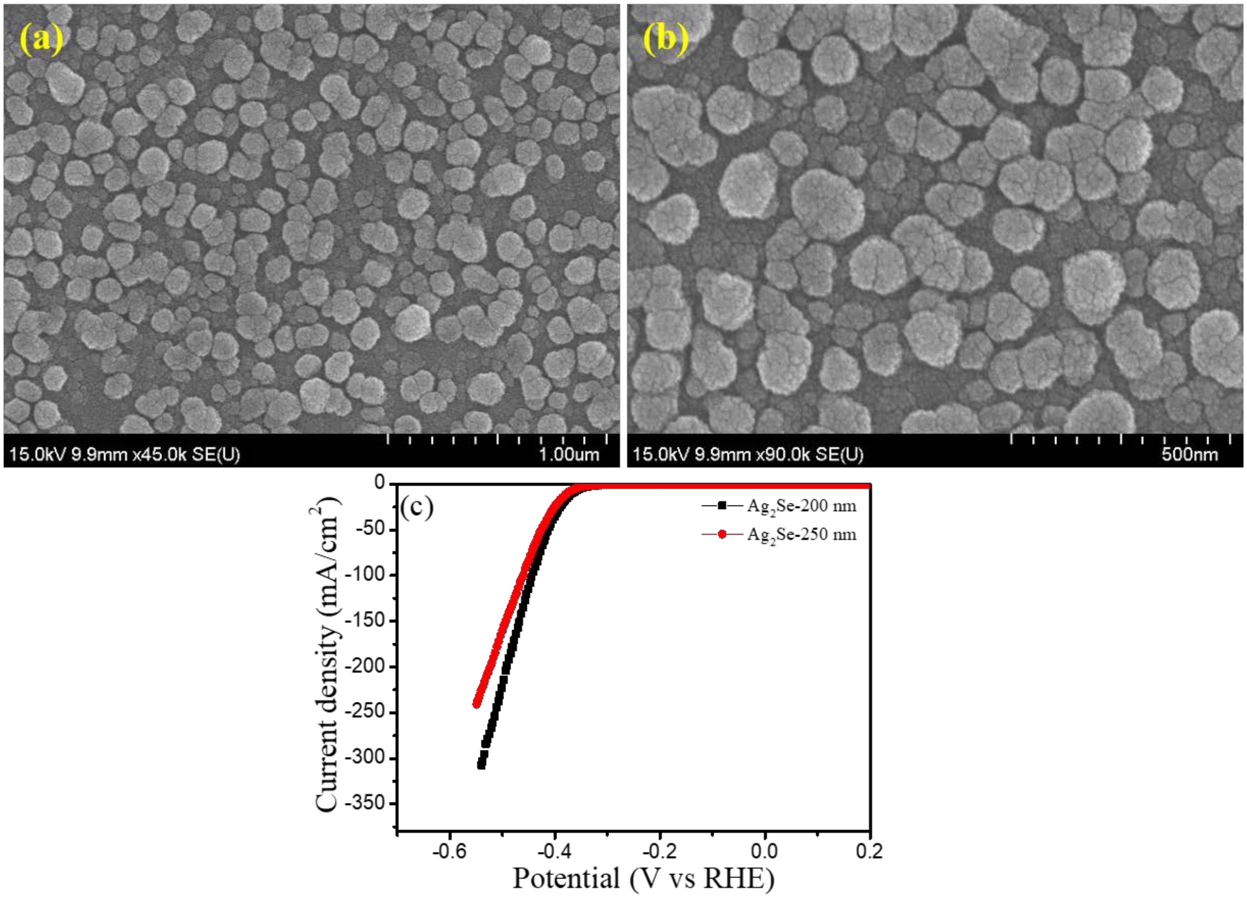

Figure S4. (a-b) FE-SEM image of Ag_2Se-250 **(c)** Polarization curves of Ag_2Se-200 and Ag_2Se-250.

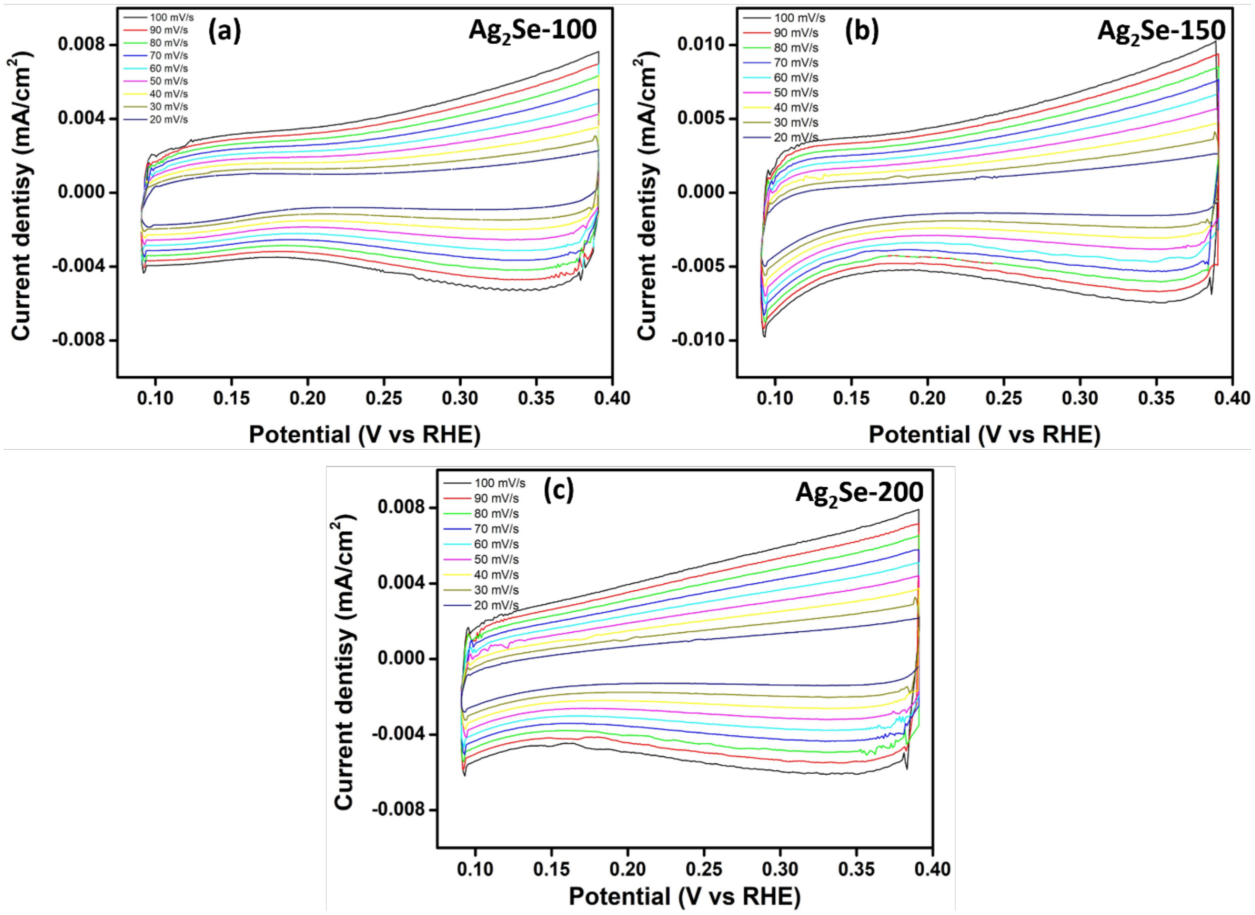

Figure S5. Electrochemical cyclic voltammetry curves of as-grown catalysts at different potential scanning rates. (a) Ag_2Se-100 (b) Ag_2Se-150 and (c) Ag_2Se-200.

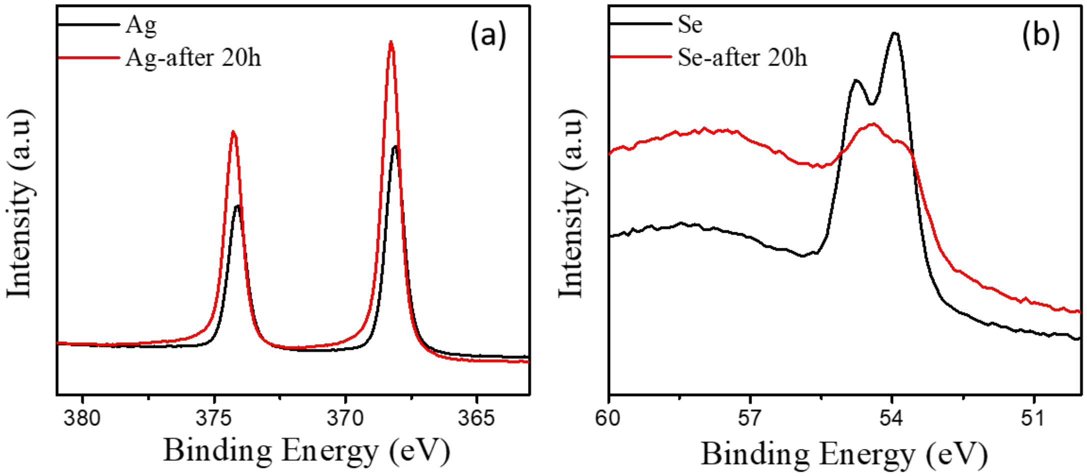

Figure S6. XPS spectra of before and after 20 h HER performance of Ag_2Se-200 (a) Ag 3d and (b) Se 2p orbitals.

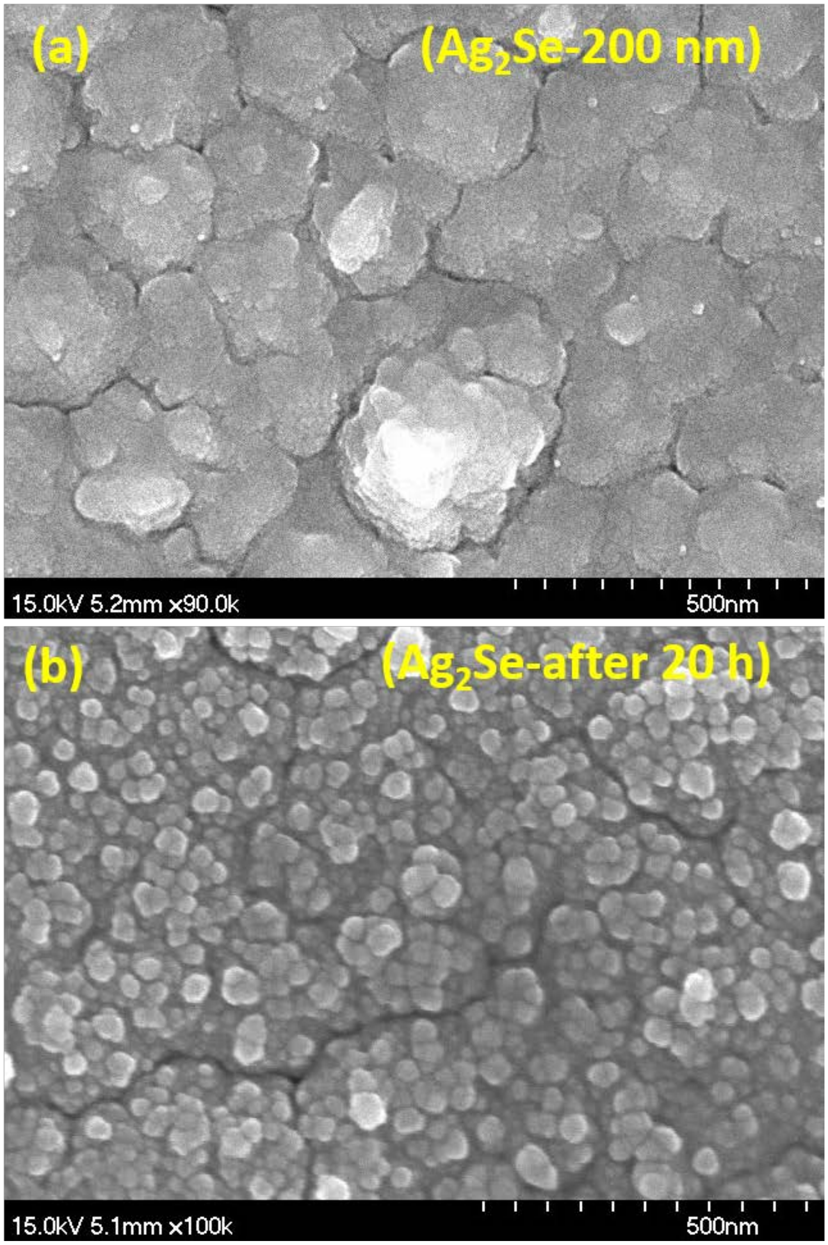

Figure S7. **(a-b)** FE-SEM image of Ag_2Se-200 catalyst before and after 20 h HER operation.

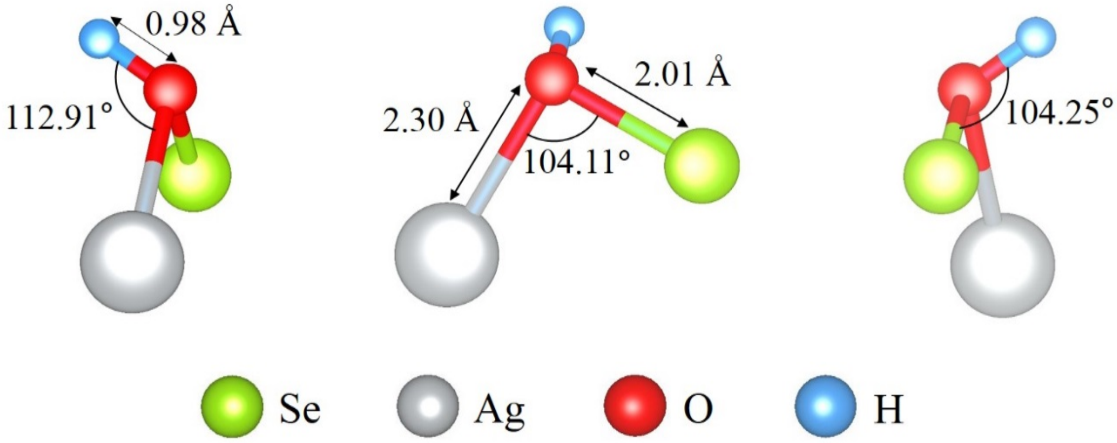

Figure S8. Geometrical bond distances for the absorption process.

Table S1. HER catalytic performances for different electrocatalysts.

**Electrocatalyst**	**Electrolyte**	**η (mV)**	**Tafel Slope ($mV \cdot dec^{-1}$)**	**j_0 ($mA \cdot cm^{-2}$)**	**Ref**
**Ag_2Se-200**	**0.5 M H_2SO_4**	**-367 @ 10 mA/cm^2**	**53**	**1.02×10^{-3}**	**This work**
Ag-Ag_2S/MoS_2	0.5 M H_2SO_4	-410 mV @ 10 mA/cm^2	-	-	[1]
1.4 Ag/Ag_2S	0.5 M H_2SO_4	-190 mV @ 10 mA/cm^2	150		[2]
Ag_2S/Ag	0.5 M H_2SO_4	-199 mV @ 10 mA/cm^2	102		[3]
Ag_2S/CuS	0.5 M H_2SO_4	-193 mV @ 10 mA/cm^2	75		[4]
$MoSe_2/Bi_2Se_3$ hybrids	0.5 M H_2SO_4	-300 @ 85 mA/cm^2	44	-	[5]
$MoSe_2/NiSe_2$ NWs	0.5 M H_2SO_4	-249 @ 100 mA/cm^2	46.9		[6]

References

1. Xia, X.; Zhao, X.; Ye, W.; Wang, C. Highly porous ag-ag2s/mos2 with additional active sites synthesized by chemical etching method for enhanced electrocatalytic hydrogen evolution. *Electrochimica Acta* **2014**, *142*, 173-181.
2. Cova, C.M.; Zuliani, A.; Santiago, A.R.P.; Caballero, A.; Muñoz-Batista, M.J.; Luque, R. Microwave-assisted preparation of ag/ag 2 s carbon hybrid structures from pig bristles as efficient her catalysts. *Journal of Materials Chemistry A* **2018**, *6*, 21516-21523.
3. Basu, M.; Nazir, R.; Mahala, C.; Fageria, P.; Chaudhary, S.; Gangopadhyay, S.; Pande, S. Ag2s/ag heterostructure: A promising electrocatalyst for the hydrogen evolution reaction. *Langmuir* **2017**, *33*, 3178-3186.
4. Ren, H.; Xu, W.; Zhu, S.; Cui, Z.; Yang, X.; Inoue, A. Synthesis and properties of nanoporous ag2s/cus catalyst for hydrogen evolution reaction. *Electrochimica Acta* **2016**, *190*, 221-228.
5. Yang, J.; Wang, C.; Ju, H.; Sun, Y.; Xing, S.; Zhu, J.; Yang, Q. Integrated quasiplane heteronanostructures of $mose_2/bi_2se_3$ hexagonal nanosheets: Synergetic electrocatalytic water splitting and enhanced supercapacitor performance. *Adv. Funct. Mater.* **2017**, *27*, 1703864.
6. Zhang, L.; Wang, T.; Sun, L.; Sun, Y.; Hu, T.; Xu, K.; Ma, F. Hydrothermal synthesis of 3d hierarchical mose 2/nise 2 composite nanowires on carbon fiber paper and their enhanced electrocatalytic activity for the hydrogen evolution reaction. *Journal of Materials Chemistry A* **2017**, *5*, 19752-19759.
